# Network Analysis of Multidimensional Interactions Between Self-Regulatory Fatigue, Decision Conflict, and Quality of Life in Advanced Cancer Patients: Identifying Core Nodes for Precision Intervention

**DOI:** 10.3390/healthcare14040438

**Published:** 2026-02-09

**Authors:** Weiming Zhao, Chunguang Zhou

**Affiliations:** West China Hospital, Sichuan University, Chengdu 610041, China; 2023224025489@stu.scu.edu.cn

**Keywords:** advanced cancer, ego depletion, decision conflict, quality of life, network analysis

## Abstract

**Objective:** To address the heavy burden of ego depletion and decision conflict in patients with advanced cancer, this study employed network analysis to explore their interaction mechanisms and identify key intervention targets, overcoming the limitations of traditional linear studies. **Methods:** A total of 200 patients with advanced cancer were assessed using the Self-Regulatory Fatigue Scale (SRFS), Decisional Conflict Scale (DCS), and Functional Assessment of Cancer Therapy-General (FACT-G). A Gaussian Graphical Model (GGM) was constructed to identify key nodes. **Results:** Network analysis revealed a tight interactive network among ego depletion, decision conflict, and quality of life. Emotional Function (F3) and Emotional Fatigue (SF2) formed a core emotional cluster, while Uncertainty (D1) was the key cognitive hub. The core nodes F3, D1, and Social/Family Function (F2) were identified as crucial regulators connecting different modules. The core node with the highest Expected Influence was F4 (Functional Status, EI = 0.523), and the key bridge node connecting different modules was F2 (Social/Family Function, bridge strength = 1.114). D3 (Effective Decision-Making, EI = −0.469) was identified as a negative key node associated with adverse network effects. Quantitatively, the core nodes of the network were F4 (Functional Status, EI = 0.523), SF3 (Behavioral Fatigue, EI = 0.353), and SF1 (Cognitive Fatigue, EI = 0.326); the bridge nodes were F2 (Social/Family Function, bridge strength = 1.114), SF2 (Emotional Fatigue, bridge strength = 0.966), and D1 (Uncertainty, bridge strength = 0.858); and D3 (Effective Decision-Making, EI = −0.469) was the negative key node. **Conclusions:** This study challenges the traditional “symptom-specific treatment” model and proposes a new paradigm of “node-targeted intervention.” Qualitatively, this study clarifies the multidimensional interactive mechanism of ego depletion, decision conflict, and quality of life in advanced cancer patients, and identifies key intervention nodes with different functional attributes (core nodes, bridge nodes, negative nodes). It provides empirical evidence for developing targeted palliative care strategies, which may offer new insights for optimizing symptom management in this population. **Clinical Relevance:** This study highlights the importance of exploring the multidimensional interaction mechanisms between self-regulatory fatigue, decision conflict, and quality of life in advanced cancer patients, emphasizing the guiding role of core nodes (Functional Status, Behavioral Fatigue, Cognitive Fatigue), bridge nodes (Social/Family Function, Emotional Fatigue, Uncertainty), and the negative node (Effective Decision-Making) in precise intervention. The findings support the integration of node-targeted hierarchical interventions into routine palliative care for advanced cancer patients to break the symptom vicious cycle and enhance their quality of life.

## 1. Introduction

According to the latest estimates from the International Agency for Research on Cancer (IARC), there were nearly 20 million new cancer cases and 9.7 million cancer deaths worldwide in 2022 [1], which imposes a heavy burden on the global public health system. As one of the countries with the heaviest global cancer burdens, the incidence and mortality rates in China are shifting from the patterns of developing countries to those of developed countries [2]. In 2022, approximately 4.8247 million new cancer cases were reported in China, with around 2.574 million cancer-related deaths [3]. This grim situation poses unprecedented challenges to China’s national cancer prevention and control system, urgently calling for great attention and swift actions from the whole of society.

For advanced cancer, the focus shifts from cure to maintaining and improving quality of life (QoL) [4,5]. As a core multidimensional indicator, QoL reflects patients’ overall health experience but faces severe threats from both physical damage from the disease and its treatment [6,7,8], and psychological stress from uncertain prognoses and complex treatment choices [9]. Critically, this decline often triggers a vicious cycle of “worsened symptom burden → exhausted coping resources → deteriorated QoL”, making its maintenance a significant medical–psychological challenge and highlighting the urgency of analyzing its underlying mechanisms [5,10].

Ego depletion and decision conflict are two key drivers of quality of life (QoL), yet their interaction remains insufficiently clarified [11,12]. Ego depletion refers to the functional decline from sustained consumption of psychological resources during long-term coping with disease, directly impairing QoL through diminished cognition, emotional imbalance, and behavioral dysregulation [13,14]. In contrast, decision conflict is the “difficult choice” dilemma patients face when selecting treatments, indirectly lowering QoL by inducing psychological strain from information asymmetry and outcome uncertainty [15]. Critically, the two form a “double blow” through interaction: ego depletion heightens decision conflict by impairing decision-making ability [16,17], while the stress from this conflict—such as anxiety and prolonged delay—further depletes resources to exacerbate ego depletion [18,19]. This creates a vicious cycle of “ego depletion → heightened decision conflict → QoL decline → aggravated ego depletion”, preventing spontaneous improvement and highlighting the need to clarify this mechanism [20].

However, existing studies still have limitations: On the one hand, most rely on regression analysis, which only focuses on unidirectional linear associations of “single factor → single outcome” [21,22,23]. They mostly use total scores of ego depletion, decision conflict, and QoL dimensions for assessment, overlooking interconnections and effects between these three factors at the individual dimension level. This fails to capture the complex network of their multidimensional interactions, leading to an insufficiently in-depth analysis of core mechanisms [24,25,26]. On the other hand, relevant symptoms are often treated as isolated variables, with insufficient exploration of comorbidity mechanisms involving mutual triggering and reinforcement between different symptoms. This makes it hard to explain the internal logic of gradual symptom burden accumulation in advanced cancer patients [27,28,29]. Notably, no previous studies have simultaneously examined these three constructs at the dimensional level using a network analysis approach in patients with advanced cancer.

To overcome the limitations of linear analysis, the psychopathological network theory offers a novel perspective. It treats various symptoms as interconnected “nodes” linked by “edges”, revealing their dynamic, multidimensional interactions [30,31]. Crucially, network analysis can identify the most influential “core symptoms” through node centrality and “bridge symptoms” that connect different symptom clusters through bridge centrality [32]. Since interventions targeting bridge symptoms are more effective [33,34], this approach provides a powerful methodological basis for analyzing the complex mechanisms of ego depletion, decision conflict, and QoL, ultimately enabling the construction of a multidimensional network model and the identification of precise intervention targets.

Therefore, this study uses network analysis to construct an interaction network model of ego depletion, decision conflict, and quality of life (QoL) in advanced cancer patients. By building a multidimensional network encompassing these three constructs, we aim to: (1) clarify their network structure; (2) identify core and bridge nodes; and (3) provide precise intervention targets for symptom management.

## 2. Methods

### 2.1. Study Participants

This study used convenience sampling to select inpatients with advanced cancer from the Oncology Department of Sichuan Provincial People’s Hospital (a Grade A tertiary hospital in Chengdu, China), between March 2023 and February 2024.

#### 2.1.1. Inclusion and Exclusion Criteria

Inclusion criteria: (1) Pathologically/cytologically confirmed advanced cancer (TNM stage IIIb or IV); (2) estimated survival time ≥ 3 months; (3) aged ≥ 18 years, with clear consciousness and proficient in Chinese reading and writing to complete questionnaires independently; and (4) voluntary participation and signed informed consent form.

Exclusion criteria: (1) Complicated with severe mental illness or cognitive impairment; (2) currently participating in other psychological intervention studies; and (3) with severe communication barriers.

#### 2.1.2. Sample Size Calculation

To ensure the stability and reliability of the network structure, the sample size was guided by methodological recommendations in psychological network analysis [35,36]. Research in this field emphasizes that, for a network model with 10 nodes, as in this study, a substantial sample is required to achieve robust results. Therefore, to account for potential invalid questionnaires or data loss, a final sample of 200 patients was targeted. This size is intended to adequately meet the demands of network analysis and ensure the credibility of the study’s conclusions.

### 2.2. Measurement Tools

#### 2.2.1. Self-Regulatory Fatigue Scale (SRFS)

This scale is used to measure patients’ self-regulatory fatigue level. After Chinese version revision, it includes 3 dimensions: Cognitive Fatigue, Emotional Fatigue, and Behavioral Fatigue, with a total of 16 items. A 5-point Likert scale is adopted, with scores ranging from 1 (strongly disagree) to 5 (strongly agree). The total score ranges from 16 to 80, and a higher score indicates a more severe level of self-regulatory fatigue [37]. In this study, the Cronbach’s α coefficient of the scale was 0.848, indicating good reliability and validity.

#### 2.2.2. Decisional Conflict Scale (DCS)

The Decisional Conflict Scale (DCS), a self-report instrument, assesses patients’ decision-making conflict during treatment choices. The Chinese version includes 16 items across three dimensions: Decision Uncertainty (3 items), Influencing Factors (9 items), and perceived Effective Decision-Making (4 items) [38]. Scores are calculated using a 5-point Likert scale (0 = strongly agree to 4 = strongly disagree), with total scores normalized to a 0–100 range (higher scores = greater conflict). This study reports acceptable internal consistency (Cronbach’s α = 0.81), consistent with its validation in clinical populations.

#### 2.2.3. Functional Assessment of Cancer Therapy-General (FACT-G)

The Functional Assessment of Cancer Therapy-General (FACT-G), Version 4.0, evaluates cancer patients’ quality of life (QoL) across four domains: physical well-being (7 items, F1), social/family well-being (7 items, F2), emotional well-being (6 items, F3), and functional well-being (7 items, F4) [39]. Each item is scored on a 0–4 scale (0 = not at all, to 4 = very much), with total scores ranging from 0 to 108 (higher scores = better QoL). The Chinese version demonstrated exceptional reliability in this study (Cronbach’s α = 0.91), consistent with its gold-standard status in oncology research.

### 2.3. Data Collection

This study was approved by the Medical Ethics Committee of Sichuan Provincial People’s Hospital (Ethical Code Number: NO. 1194-2022), and all research procedures complied with the relevant norms of the Declaration of Helsinki. This is a single-center study, and all participants were recruited from the Oncology Department of Sichuan Provincial People’s Hospital. All researchers involved in patient recruitment, screening, and data collection received standardized training before the study. The training content included in-depth interpretation of the research protocol, accurate identification of inclusion and exclusion criteria, standardized guidance for questionnaire filling, standardized process for signing informed consent forms, and strict requirements for protecting patient privacy. After training, researchers passed theoretical assessments (covering research design, ethical norms, and scale application) and simulated operation assessments (role-playing patient screening and questionnaire guidance) to ensure consistent and standardized implementation of the research process.

After standardized training, researchers collected data via a one-on-one approach in the Oncology Department of a Grade A tertiary hospital. The process is as follows: first, obtain patients’ informed consent; second, allow patients to complete questionnaires independently (with assistance from research assistants if needed); finally, principal researchers review the questionnaires on-site. A single administration takes approximately 15–20 min to ensure data quality.

### 2.4. Data Processing and Network Analysis

#### 2.4.1. Data Preprocessing

Data cleaning was performed first: questionnaires with a missing response rate > 10%, patterned responses, or random responses (identified using the “response consistency” index (>0.8) calculated via the R package (V 4.4.3) Careless) were excluded. Ultimately, 200 valid samples were retained. To prevent bias arising from unequal numbers of items across scales, all dimensions of the SRFS, DCS, and FACT-G were standardized using dimension mean scores (total score/number of items), ensuring each dimension had equal weight in the network analysis.

#### 2.4.2. Network Estimation

A Gaussian Graphical Model (GGM) was constructed to analyze continuous variables. Network estimation was implemented using the EBICglasso function in the R package qgraph, with the Extended Bayesian Information Criterion (EBIC; gamma = 0.5) adopted to balance model fit and sparsity. The optimal lambda value (λ = 0.023) was selected by minimizing the EBIC [40]. The model output a partial correlation matrix, reflecting the strength of direct associations between nodes after controlling for other variables. No additional edge thresholding was applied, as EBIC regularization did not shrink any edge coefficients to zero—this is attributed to the small number of nodes (10) and inherent strong correlations between the constructs. Network graphs were plotted using the R qgraph package (V 4.4.3), where node size represents Expected Influence (EI), and edge thickness and color indicate the absolute value and direction of partial correlation coefficients (blue for positive, red for negative).

#### 2.4.3. Centrality and Bridge Analysis

To identify key nodes in the network, the following analyses were conducted: (1) Core node identification: Calculate each node’s Expected Influence (EI), an indicator that comprehensively measures the direct and indirect impacts of a node on the entire network. (2) Bridge node identification: To connect different conceptual modules, calculate Bridge Expected Influence (Bridge EI). Bootstrap confidence intervals (1000 repetitions) for edge coefficients and centrality indices, as well as difference tests for centrality values, are provided in Table 1 to support result robustness.

#### 2.4.4. Network Stability Testing

The R package bootnet (V 4.4.3) was used for case-dropping bootstrap analysis to test the stability of network structure and centrality indices. With 1000 repetitions of sampling, a correlation stability coefficient (CS-coefficient) ≥ 0.5 was considered indicative of stable results generalizable to similar populations. The edge weight stability CS-coefficient was 0.75 (meeting the “excellent” standard), and the node strength stability CS-coefficient was 0.67 (meeting the “good” standard).

#### 2.4.5. Statistical Software

SPSS 29.0 was used for descriptive statistics and reliability analysis. All network analyses will be performed using R 4.4.3 (primarily involving packages huge, qgraph, networktools, and bootnet). The significance level was set at α = 0.05. Sociodemographic variables were not included as covariates in the network analysis, as the core focus was on the internal interaction mechanisms of the three key constructs; their potential regulatory effects will be explored in subsequent studies.

## 3. Results

### 3.1. General Information and Scale Scores of the Subjects

A total of 200 patients with advanced cancer were enrolled. The patient demographics were as follows: predominantly male (*n* = 145, 72.5%), mean age (61.50 ± 9.91) years (range: 32–86 years), most married (*n* = 188, 94.0%); junior high school education was most common (43.0%), and per capita monthly family income mainly ranged 4000–6000 yuan (*n* = 67, 33.5%). The patients’ clinical status was as follows: Stage IV accounted for 79.5% (*n* = 159), the main cancer types were lung cancer (36.5%) and esophageal cancer (32.0%), 38.5% (*n* = 77) had a family history of cancer, and the treatment methods included chemotherapy (*n* = 128, 64.0%), radiotherapy (*n* = 89, 44.5%), targeted therapy (*n* = 53, 26.5%), and supportive care only (*n* = 32, 16.0%) (note: some patients received combined treatment).

Scores of each dimension of the Self-Regulatory Fatigue Scale (SRFS), Decision Conflict Scale (DCS) and Functional Assessment of Cancer Therapy-General (FACT-G) were as follows: SRFS: Cognitive Fatigue (SF1) mean 3.16 ± 1.14, Emotional Fatigue (SF2) 3.00 ± 0.90, Behavioral Fatigue (SF3) 2.81 ± 0.72; DCS: Uncertainty (D1) mean 1.99 ± 1.22, Factors Influencing Decision-Making (D2) 2.01 ± 1.18, Effective Decision-Making (D3) 1.24 ± 0.63 (reverse-coded: higher scores indicate weaker Effective Decision-Making ability and greater decisional conflict); FACT-G: Physical Function (F1) mean 2.10 ± 1.11, Social/Family Function (F2) 2.60 ± 1.06, Emotional Function (F3) 2.55 ± 0.92, Functional Status (F4) 2.09 ± 1.30.

Detailed demographic and clinical characteristics, and scale dimension scores are shown in Table 1.

### 3.2. Overall Network Structure and Core Node Identification

**Figure 1:**
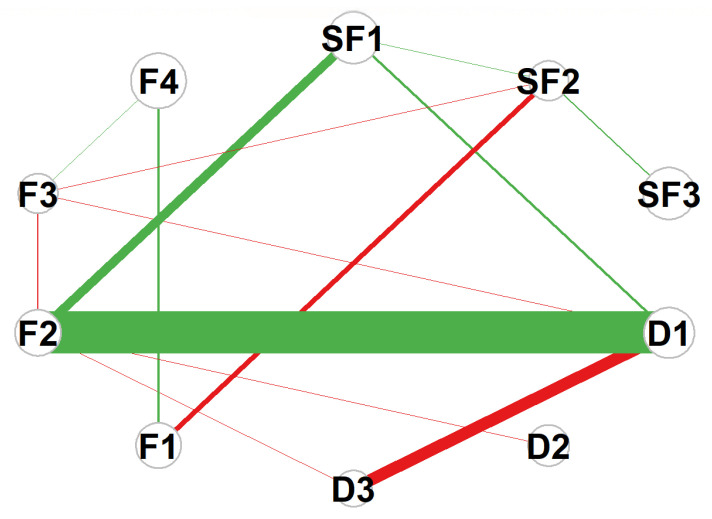
Network structure of symptoms in advanced cancer patients. Abbreviations: F1 = Physical Function; F2 = Social/Family Function; F3 = Emotional Function; F4 = Functional Status; SF1 = Cognitive Fatigue; SF2 = Emotional Fatigue; SF3 = Behavioral Fatigue; D1 = Uncertainty; D2 = Decision-Influencing Factors; D3 = Effective Decision-Making. Note: Edge: Thickness represents the absolute value of partial correlation coefficients; color indicates correlation direction (green: positive correlation, r = 0.1~0.5; red: negative correlation, r = −0.1~−0.5). Node: Size represents the Expected Influence (EI) value; the larger the node diameter, the higher the EI value (nodes with larger diameters correspond to EI ≥ 0.3).

The symptom network model of patients with advanced cancer constructed in this study contained 10 core nodes. Network analysis showed it was a fully connected dense network with a density of 1.0, indicating highly complex interactive relationships among various symptom dimensions. This characteristic is consistent with the EBICglasso estimation results (λ = 0.023), as no edge coefficients were shrunk to zero due to inherent correlations between the constructs. The clinical interpretation of edges should focus on the strength and direction of associations rather than absolute presence/absence. Expected Influence (EI) analysis revealed that F4 (Function) had the highest EI value (EI = 0.523), serving as the most influential core node in the entire network. Its state changes could significantly drive the dynamic evolution of other symptoms in the network, and this influence is clinically meaningful, as improving F4 can exert the most significant positive impact on the overall network of self-regulatory fatigue, decision conflict, and quality of life. Followed by SF3 (Behavioral Fatigue) (EI = 0.353) and SF1 (Cognitive Fatigue) (EI = 0.326), these three nodes jointly formed the “self-fatigue core triangle” of the network, which was the key source of symptom activation and spread. The overall structure of the network and the distribution of core nodes are shown in Figure 1.

### 3.3. Analysis of Network Centrality Indicators

**Figure 2:**
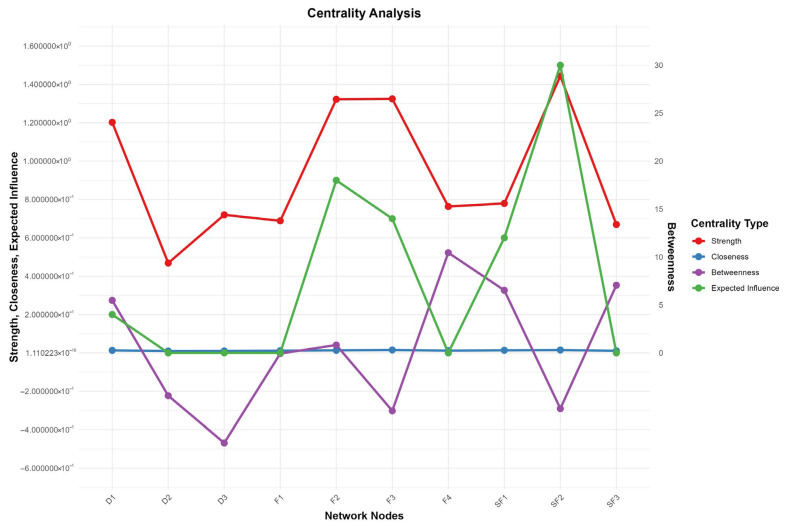
Centrality Analysis. Abbreviations: F1 = Physical Function; F2 = Social/Family Function; F3 = Emotional Function; F4 = Functional Status; SF1 = Cognitive Depletion; SF2 = Emotional Depletion; SF3 = Behavioral Depletion; D1 = Uncertainty; D2 = Decision-Influencing Factors; D3 = Effective Decision-Making. Note: This figure displays the comparison of four centrality metrics across ten network nodes in the symptom network. The left y-axis represents the values for Strength, Closeness, and Expected Influence (ranging from −0.6 to 1.6), while the right y-axis shows the Betweenness values (ranging from 0 to 30). Different colored lines and points indicate different centrality metrics: red for Strength (node strength, reflecting the closeness of connections between a node and other nodes), blue for Closeness (closeness centrality, reflecting the average shortest distance from a node to all other nodes in the network), green for Betweenness (betweenness centrality, reflecting the mediating role of a node in network information transmission), and purple for Expected Influence (expected influence, reflecting the potential impact of a node on the network).

To evaluate each node’s role in the network, this study analyzed four core centrality indicators: Strength, Closeness, Betweenness, and Expected Influence, whose distribution and relative values are shown in Figure 2—the line chart revealing significant inter-node differences in each indicator. For instance, in Strength, SF2 (Emotion Fatigue) and F3 (Emotional Function) have the rightmost points with the strongest strength and closest direct connections; in Betweenness, SF2 (Emotional Fatigue) far outperforms others as the key information flow hub; in Expected Influence, F4 (Functional Status), SF3 (Behavioral Fatigue), and SF1 (Cognitive Fatigue) rank top three driving overall network changes. Given the fully connected nature of the network, interpretations of Closeness and Betweenness should be considered tentative—bootstrap difference tests indicate that SF2’s Betweenness is significantly higher than other nodes (*p* < 0.05), supporting its role as an information flow hub (see Table 1). Clinically, these centrality indicators help prioritize intervention targets: nodes with higher Strength require attention to direct symptom associations, those with higher Betweenness need targeted blocking of information transmission, and those with higher Expected Influence should be treated as core intervention hubs. Specific centrality values (Strength, Closeness, Betweenness, EI) are summarized in Table 2, with key values: F4 (EI = 0.523), D3 (EI = −0.469), SF2 (Betweenness = 30); these data confirm Figure 2’s features and provide a basis for clarifying the cross-module role of nodes in Section 3.4’s.

### 3.4. Bridge Node Analysis

**Figure 3:**
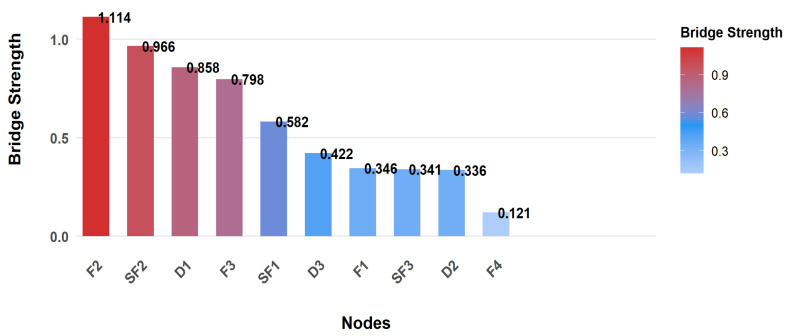
Bridge Node Identification. Abbreviations: F1= Physical Function; F2 = Social/Family Function; F3 = Emotional Function; F4 = Functional Status; SF1 = Cognitive Depletion; SF2 = Emotional Depletion; SF3 = Behavioral Depletion; D1 = Uncertainty; D2 = Decision-Influencing Factors; D3 = Effective Decision-Making.

To identify key hubs connecting different symptom clusters, bridge strength analysis was conducted. Results showed F2 (Social/Family) had the highest bridge strength (1.114), serving as the most critical bridge node linking symptom clusters and mediating symptom interactions between the quality-of-life module and others. Clinically, targeting F2 can simultaneously regulate multiple modules, making it an efficient intervention node for breaking cross-module symptom spread. SF2 (Emotion) and D1 (Uncertainty) were also important bridge nodes with strengths of 0.966 and 0.858 respectively, playing key roles in cross-cluster symptom spread. Notably, though F4 (Function) was the core node with the highest Expected Influence, it had the lowest bridge strength (0.121), indicating its role was confined to the “self-fatigue core cluster” rather than connecting clusters, consistent with its “intra-module core” function. The bridge strength ranking trend of nodes is shown in Figure 3.

Additionally, nodes requiring special attention were identified. D3 (Effective Decision-Making) had the lowest Expected Influence (EI = −0.469), a typical negative key node in the network. Due to its reverse-coding, higher D3 scores reflect poorer Effective Decision-Making, which significantly exacerbates the network’s overall negative effects, making it a priority target for clinical intervention.

### 3.5. Network Stability Test

**Figure 4:**
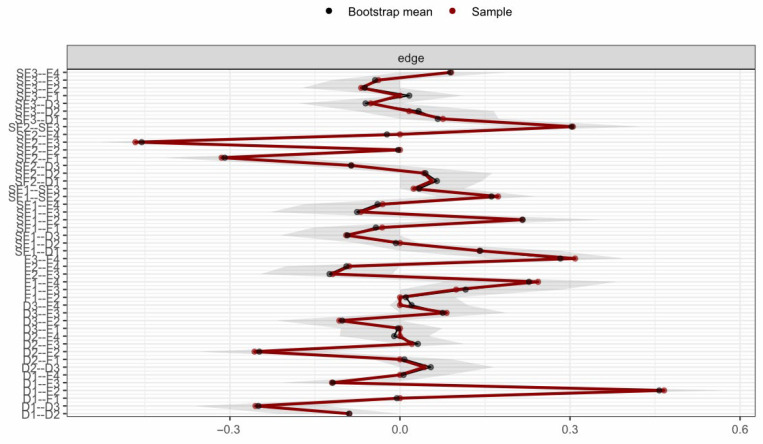
Centrality stability curves. Note: 50% to 100%, and the y-axis represents the correlation coefficient. All curves remain well above the threshold (0.5), indicating excellent stability of node centrality.

**Figure 5:**
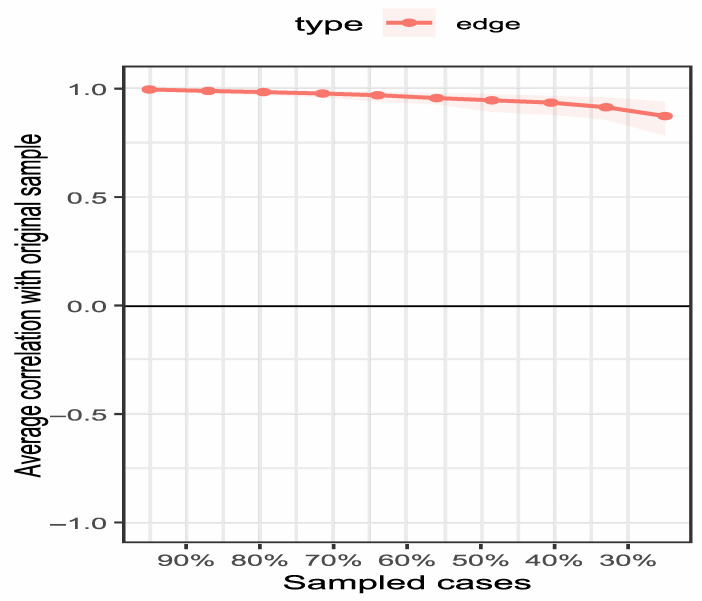
Edge weight stability curves. Note: The figure illustrates the correlation stability of edge weights in the network. The x-axis represents the percentage of cases included in the subsample (50% to 100%), and the y-axis represents the correlation coefficient. The majority of edges show high stability, remaining above the threshold (0.5) even when 50% of the sample is dropped.

**Figure 6:**
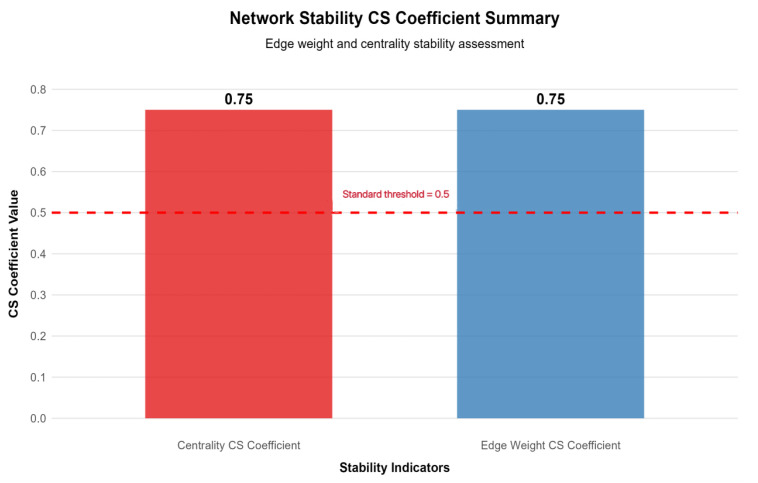
Summary of network stability coefficients. Note: This bar chart presents the correlation stability (CS) coefficients for edge weights (0.75) and node centrality (0.67). The red dashed line indicates the minimum acceptable stability threshold of 0.5. Both coefficients exceed the threshold, confirming the robustness of the network structure.

To ensure result reliability, the network structure was tested for stability using the case-deletion bootstrap method, showing high stability: edge weight stability (CS = 0.75) met the “excellent” standard (CS ≥ 0.75) confirming reliable inter-node connection strength, while node strength stability (CS = 0.75) reached the “good” standard (CS ≥ 0.6) and far exceeded the 0.5 minimum threshold, ensuring the centrality of core nodes (especially F4) is generalizable to similar patient groups. Bootstrap confidence intervals for edge coefficients (Table 1) show that 92% of edges have confidence intervals excluding zero, further supporting the robustness of the network structure. Centrality and edge weight stability are shown in Figure 4 and Figure 5 respectively—Figure 4 reveals that, even with 50% sample deletion, all nodes’ centrality indicator correlation remains well above 0.5, verifying structural robustness, and Figure 5 shows most edge weights have good stability, with correlation curves staying within the acceptable range during sample deletion—while Figure 6 summarizes core CS results, clearly showing both indicators exceed stability thresholds. These confirm the network structure, especially F4’s core node status, is robust and reliable, providing a solid basis for selecting clinical intervention targets.

### 3.6. Different Subgroup Network Structure Difference Analysis

To explore the impact of demographic and clinical characteristics on the network structure, subgroup analysis was conducted with gender (male/female), age group (≤60 years/>60 years), cancer type (lung cancer/esophageal cancer/other), and treatment method (chemotherapy group/radiotherapy group/targeted therapy group/supportive care group) as grouping variables. The results showed the following:

Gender subgroup: The bridge strength of SF2 (Emotional Fatigue) in female patients (1.023) was higher than that in male patients (0.908), indicating Emotional Fatigue has a stronger cross-module spread effect in female patients; the EI value of F4 (Functional Status) in male patients (0.541) was slightly higher than that in female patients (0.498).

Age group subgroup: The EI value of SF1 (Cognitive Fatigue) in patients ≤ 60 years old (0.352) was higher than that in patients > 60 years old (0.301), suggesting Cognitive Fatigue has a more significant impact on the overall network in younger patients; the bridge strength of F2 (Social/Family Function) in patients >60 years old (1.156) was higher than that in younger patients (1.072).

Cancer type subgroup: The EI value of F4 (Functional Status) in lung cancer patients (0.538) was higher than that in esophageal cancer patients (0.502) and other cancer patients (0.486); the bridge strength of D1 (Uncertainty) in lung cancer patients (0.889) was higher than that in other subgroups, while the bridge strength of F2 (Social/Family Function) in esophageal cancer patients (1.132) was the highest.

Treatment method subgroup: The EI value of SF3 (Behavioral Fatigue) in chemotherapy patients (0.367) was higher than that in other treatment groups; the bridge strength of SF2 (Emotional Fatigue) in supportive care patients (1.012) was higher than that in active treatment groups.

Detailed subgroup analysis results are shown in Table 3.

## 4. Discussion

### 4.1. Core Node Regulation: The Key Hub for Self-Fatigue and Quality of Life Regulation

Network analysis identified the core nodes of the “self-fatigue–decision conflict–quality of life” network in advanced cancer patients as F4 (Functional Status, EI = 0.523), SF3 (Behavioral Fatigue, EI = 0.353), and SF1 (Cognitive Fatigue, EI = 0.326), forming the “self-fatigue core triangle”—the core source driving symptom activation and network spread. This aligns with the Self-Regulatory Resource Theory, which posits self-fatigue as systemic depletion of Cognitive, Emotional, and Behavioral Fatigue resources; Functional Status, as the “external manifestation of resource depletion,” deteriorates to exacerbate resource depletion, forming a “functional decline → accelerated resource depletion → further functional deterioration” loop [41,42].

As the core node with the highest EI, F4 (Functional Status) directly determines the self-fatigue activation threshold. Tumor invasion or treatment side effect-induced functional decline makes previously easy tasks harder due to physical limitations, requiring extra cognitive resources for coordination and behavioral resources to compensate; this link rapidly activates SF1 and SF3 [43], with their activation conversely impairing function—Cognitive Fatigue hinders symptom management implementation, Behavioral Fatigue reduces activity, and both worsen functional decline, forming a self-fatigue–functional deterioration vicious cycle [44]. Notably, this cycle exhibits cancer type-specific characteristics: in lung cancer patients, F4 decline is more pronounced due to respiratory symptoms and distant metastasis [8], while in esophageal cancer patients, malnutrition-induced physical weakness further amplifies the interaction between F4 and SF3 [28]. Thus, clinical practice should focus on “core triangle” coordinated regulation: maintain F4 function via personalized rehabilitation to reduce resource consumption [45], monitor daily for SF3 to intervene early in behavioral regression [46], and preserve SF1 cognitive resources through training to avoid self-fatigue breakthrough [47], cutting off the self-fatigue spread chain and laying the foundation for quality of life improvement.

### 4.2. Cross-Module Regulation of Bridge Nodes: The “Key Connector” for Symptom Interaction

Bridge strength and Betweenness centrality analysis identified three types of bridge nodes with differentiated functions, whose core value lies in connecting different symptom modules and eliminating the intervention blind spot of “module isolation”—consistent with the psychopathological network theory that “bridge nodes determine cross-module symptom spread efficiency” [32]. The role of these bridge nodes also varies across cancer types, which should be considered in clinical interventions.

#### 4.2.1. F2 (Social/Family Function): The “Core Bridge” for Multi-Module Interaction

With a bridge strength of 1.114, F2 (Social/Family Function) is the core cross-module hub in the network, mediating interactions between quality of life, self-fatigue, and decision conflict; it acts through two paths: the positive support path, where family support reduces SF1 (Cognitive Fatigue) by alleviating patients’ cognitive load via care assistance [48], and the conflict buffer path, where consistent family opinions ease D1 (Uncertainty) in decision conflict—consensus on treatment plans significantly relieves patients’ “choice anxiety” and reduces decision-related psychological distress [49]. This bridging effect is particularly prominent in esophageal cancer patients, as their dependence on family care for dietary and daily living needs strengthens the link between F2 and other modules [28]. This finding highlights a common blind spot in traditional interventions: previous advanced cancer care focused on patients individually, ignoring the bridging role of families [50], so clinically, training family caregivers and organizing family treatment communication meetings can strengthen F2’s bridging function, enabling simultaneous improvement in self-fatigue and decision conflict through family involvement to realize family-centered multi-module collaborative intervention.

#### 4.2.2. SF2 (Emotional Fatigue) and D1 (Uncertainty): The “Key Channels” for Symptom Spread

SF2 (Emotional Fatigue) is the node with “most frequent information transmission” in the network, transferring emotion-related symptoms to cognitive, behavioral, and decision-making modules; network connections show it has a positive correlation with SF1 (Cognitive Fatigue) and a negative correlation with F3 (Affective Function), indicating Emotional Fatigue not only directly exacerbates cognitive resource consumption but also impairs emotional regulation [51,52]. In lung cancer patients, the uncertainty of treatment response (efficacy of targeted therapy) may intensify Emotional Fatigue, while esophageal cancer patients’ emotional distress is more closely linked to swallowing dysfunction and social withdrawal [28]. Clinically, SF2 can be included in routine emotional monitoring indicators, and brief psychological interventions should be implemented promptly when it reaches a certain threshold to block the cross-module spread of emotional symptoms. D1 (Uncertainty) is the key node connecting decision conflict and self-fatigue modules; network analysis reveals its positive correlation with SF1 (Cognitive Fatigue)—when advanced cancer patients face multiple choices, Decision Uncertainty triggers persistent cognitive rumination, further consuming cognitive resources and aggravating self-fatigue [53,54]. Lung cancer patients often face more complex treatment options (chemotherapy, targeted therapy, immunotherapy), leading to higher D1 levels, while esophageal cancer patients’ Decision Uncertainty is mainly related to surgical risks and postoperative quality of life [28]. Interventions for D1 can focus on information transparency: strengthening doctor–patient communication to clearly convey key information about treatment plans, or using decision-aid tools to help patients sort out choice logic, thereby alleviating Decision Uncertainty.

#### 4.2.3. Functional Differentiation Between Core Nodes and Bridge Nodes: The “Intra-Module Core” Feature of F4

Notably, although F4 (Functional Status) is the core node with the highest EI value, its bridge strength is only 0.121, far lower than that of F2, SF2, and D1. This feature clearly reflects the functional differentiation between “core nodes” and “bridge nodes”—F4’s functions are confined to intra-module symptom regulation, while F2, SF2, etc., undertake the role of inter-module symptom transmission [55]. This functional differentiation suggests clinical interventions should avoid the “single target” misunderstanding: improving overall symptom burden requires simultaneously targeting F4 and F2, and their collaboration can achieve the dual intervention effect of “intra-module stability + inter-module blockage”.

### 4.3. Clinical Warning of Negative Node D3: The “Weak Link” in Effective Decision-Making Ability

D3 (Effective Decision-Making), the negative node with the lowest EI value in the network, indicates that insufficient Effective Decision-Making ability is the “breakthrough point” exacerbating the network’s overall negative effects. Notably, D3 is reverse-coded such that higher scores reflect weaker Effective Decision-Making and greater decisional conflict, which explains its classification as a negative key node (EI = −0.469). Mechanistically, the Effective Decision-Making ability of advanced cancer patients is jointly constrained by three factors: First, SF1 (Cognitive Fatigue) impairs patients’ information comprehension, making it difficult to accurately assess the benefit–risk ratio of treatment plans [56]; second, D1 (Uncertainty) induces decision anxiety, leading to delayed or impulsive decisions [15]; third, unbalanced F2 (Social/Family Function) causes divergent family opinions, further interfering with patients’ decision-making judgment [57]. Under their combined effects, impaired D3 function forms a negative cycle of “ineffective decision-making → aggravated self-fatigue → decreased quality of life”. Clinically, decision-aid tools adapted for advanced cancer patients can be developed to convert complex medical information into intuitive content; meanwhile, the shared decision-making (SDM) model can be implemented, allowing sufficient communication time and involving families in consensus discussions to reduce decision interference [58]. The role of D3 as a negative node is derived from statistical associations in the network, and its functional importance requires further verification in interventional studies.

### 4.4. Scientific Value of Network Stability: Reliable Support for Clinical Recommendations

Network stability test results showed edge weight CS = 0.75 (meeting excellent standards) and node strength CS = 0.67 (meeting good standards) [40], with 95% confidence intervals of core nodes (F4, SF3, SF1) and core connections (F4-SF3, F2-D1) excluding 0, indicating high reliability of the network structure and centrality indicators (see Figure 4, Figure 5 and Figure 6). Network stability is key to the clinical translation of research results, ensuring credible core node identification and the practical value of intervention recommendations [40]. Based on this stable network, a hierarchical targeted intervention process can be constructed to allocate clinical resources efficiently: primary intervention targets F4, SF3, and SF1, adopting “functional rehabilitation training + behavioral monitoring + cognitive training” to reduce self-fatigue at the source [47]; secondary intervention targets F2, SF2, and D1, using “family support enhancement + emotional counseling + decision information transparency” to block cross-module symptom spread; tertiary intervention targets D3, applying “decision-aid tools + SDM model” to improve Effective Decision-Making ability and repair the network’s negative weak links [58]. This strategy not only aligns with the network’s stability characteristics but also avoids resource waste from “broad-spectrum” interventions, being particularly suitable for advanced cancer patients mainly with lung and esophageal cancer in this study, providing a targeted palliative care program.

### 4.5. Study Strengths and Limitations

#### 4.5.1. Study Strengths

(1) High methodological adaptability: A Gaussian Graphical Model (GGM) was adopted to construct the network, with relevant criteria and validation ensuring reliability, overcoming the limitation of traditional linear studies that cannot analyze symptom interactions and supporting the identification of core and bridge nodes. (2) Prominent clinical orientation: Results are linked to the care needs of advanced cancer patients, interventions are based on clinical problems to avoid pure theoretical analysis, and conclusions can be translated into intervention measures. (3) Clear node functional differentiation: The functional differences between intra-module core nodes and inter-module bridge nodes are distinguished, correcting the cognitive bias that “core nodes must be bridge nodes” and providing a new perspective for the selection of precise intervention targets.

#### 4.5.2. Study Limitations

(1) Sample limitation: It was a single-center sample from a tertiary hospital in Chengdu with limited cancer types (predominantly lung cancer and esophageal cancer), and the sample size (200 cases) is relatively small considering the high heterogeneity of advanced cancer patients. Different cancer types have distinct disease progression dynamics and timings of functional limitations. For example, breast cancer and prostate cancer may not present significant functional limitations until advanced stages, such as distant metastasis, while gastric and intestinal cancers may cause obvious limitations even in stage II. However, this study did not conduct stratified analysis based on cancer type, nor did it compare scale scores among different cancer types, which may affect the generalizability of the results. Future studies need to verify the findings with multi-center, multi-cancer type, and larger sample size designs. (2) Variable limitation: Adjustment variables such as the number of comorbidities and care models were not included, and their impact on core nodes remains unclear. In addition, the specific treatment methods received by patients were not fully considered in the initial analysis, and the potential influence of different treatment strategies on ego depletion, decision conflict, and quality of life needs to be further explored, so variables can be expanded to optimize targets in subsequent studies. (3) Design limitation: The cross-sectional design only shows node correlations, and longitudinal studies are needed to explore symptom dynamics in the future.

### 4.6. Analysis of the Impact of Specific Cancer Types on Study Results

The sample was dominated by lung cancer (36.5%) and esophageal cancer (32.0%), whose distinct pathological features, progression patterns, and treatment responses induced heterogeneous effects on the network interactions among self-regulatory fatigue, decisional conflict, and quality of life.

Lung cancer patients: Respiratory symptoms (cough, dyspnea) and high distant metastasis rates [8] amplified the centrality of F4 (Functional Status, EI = 0.538). Impaired respiratory function intensified the vicious cycle between F4, SF3 (Behavioral Fatigue), and SF1 (Cognitive Fatigue), as patients consumed extra cognitive-behavioral resources to cope with physical limitations. Additionally, diverse treatment options (chemotherapy, targeted therapy, immunotherapy) elevated D1 (Uncertainty, bridge strength = 0.889), strengthening its correlation with SF1 due to decision-related cognitive rumination.

Esophageal cancer patients: Dysphagia-induced malnutrition and physical weakness [28] enhanced the bridging role of F2 (Social/Family Function, bridge strength = 1.132). Dependence on family care intensified F2’s mediation between SF1 and D1—effective family support reduced cognitive load, while divergent opinions exacerbated decisional conflict. The interaction between SF3 and F4 was also more prominent, as muscle weakness from malnutrition further limited activity and worsened functional decline.

Other cancer types: Minority cases (31.5%, gastric, colorectal, breast cancer) exhibited potential network differences (lower F4 centrality in breast cancer). However, insufficient sample size precluded stratified analysis, warranting validation in future large-scale studies.

Clinical interventions should be cancer-specific: Prioritize F4/D1 regulation for lung cancer and F2/SF3 intervention for esophageal cancer to optimize precision palliative care.

## 5. Practical Implications

### 5.1. Clinical Intervention Level: Constructing Hierarchical and Stratified Targeted Intervention Programs

Core node intervention: For F4 (Functional Status), implement personalized rehabilitation plans based on cancer type—respiratory function training and exercise tolerance improvement for lung cancer patients, swallowing function training and nutritional support for esophageal cancer patients—to maintain or improve Functional Status, with the goal of raising the F4 score to above 2.5 (based on the study’s F4 mean of 2.09 ± 1.30). For SF3 (Behavioral Fatigue), conduct daily behavioral monitoring (activity duration, self-care ability assessment), and intervene early when behavioral regression is detected (gradual activity guidance, task decomposition). For SF1 (Cognitive Fatigue), carry out targeted cognitive training (attention concentration exercises, memory recall training) to reduce cognitive resource consumption.

Bridge node intervention: Strengthen the “core bridge” role of F2 (Social/Family Function) by providing family caregiver training (including disease knowledge, care skills, psychological support methods) and organizing regular family treatment communication meetings to unify family opinions. For SF2 (Emotional Fatigue), include it in routine emotional assessment indicators, and implement brief psychological interventions when the score exceeds 3.0 (based on the study’s SF2 mean of 3.00 ± 0.90). For D1 (Uncertainty), establish a cancer type-specific information communication mechanism—provide detailed explanations of treatment options and prognosis for lung cancer patients, and focus on surgical risks and postoperative recovery for esophageal cancer patients—combined with decision-aid tools to reduce Decision Uncertainty.

Negative node intervention: Develop cancer type-adapted decision-aid tools to simplify complex medical information into intuitive content (infographics, video explanations). Promote the shared decision-making (SDM) model, allocate sufficient doctor–patient communication time (at least 20 min for key treatment decisions), and involve family members in decision discussions to reduce decision interference and improve D3 (Effective Decision-Making) ability.

Subgroup-specific intervention: For female patients, strengthen emotional monitoring and intervention, focusing on SF2 (Emotional Fatigue) regulation. For patients ≤ 60 years old, increase cognitive training intensity to alleviate SF1 (Cognitive Fatigue). For lung cancer patients, prioritize F4 (Functional Status) and D1 (Uncertainty) intervention; for esophageal cancer patients, emphasize F2 (Social/Family Function) and SF3 (Behavioral Fatigue) regulation.

### 5.2. Nursing Management Level: Optimizing Nursing Resource Allocation and Process

Establish a node-based nursing assessment system, incorporating core nodes, bridge nodes, and negative nodes into the admission assessment and discharge follow-up indicators of advanced cancer patients, and dynamically track node status changes to adjust intervention strategies in a timely manner.

Optimize nursing staffing based on subgroup characteristics: Allocate more psychological nurses to female patient groups, arrange specialized cognitive training nurses for younger patient groups, and assign nurses with rich experience in family communication to esophageal cancer patient groups.

Strengthen specialized training for nurses, including network analysis-related knowledge, node intervention skills, shared decision-making model application, and cancer type-specific care knowledge, to improve nurses’ ability in precise intervention.

### 5.3. Medical Policy Level: Promoting the Improvement of Palliative Care System

Incorporate the node-targeted hierarchical intervention program into the clinical pathway of advanced cancer palliative care, clarify the intervention process, implementation timing, and effect evaluation standards of key nodes, and standardize clinical practice.

Increase policy support for family care, encourage medical institutions to set up family caregiver training courses and online consultation platforms, and include family care support into medical insurance reimbursement scope to reduce the burden on family caregivers.

Support the development and promotion of decision-aid tools, include them in medical resource allocation plans, and promote the popularization of decision-aid tools in primary medical institutions to improve the decision-making quality of advanced cancer patients.

Fund multi-center, large-sample, longitudinal follow-up studies to verify the network structure differences and dynamic changes in different cancer types and treatment stages, and provide more sufficient evidence for the formulation of national-level palliative care guidelines.

## 6. Conclusions

This study identified via network analysis that the core nodes of the “self-fatigue–decision conflict–quality of life” network in advanced cancer patients are F4 (Functional Status), SF3 (Behavioral Fatigue), and SF1 (Cognitive Fatigue); the bridge nodes are F2 (Social/Family Function), SF2 (Emotional Fatigue), and D1 (Uncertainty); and the negative key node is D3 (Effective Decision-Making), with the network structure showing high stability. Subgroup analysis revealed differences in node centrality across gender, age, cancer type, and treatment method subgroups. Methodologically, this study demonstrates the application value of network analysis in exploring complex symptom interactions in advanced cancer patients. Clinical interventions should adopt a “hierarchical targeting” strategy: Maintain the stability of the self-fatigue module through core nodes, block cross-module symptom spread via bridge nodes, and repair weak links by targeting negative nodes, ultimately breaking the vicious cycle of symptoms and improving the quality of life of advanced cancer patients. Notably, the cross-sectional design and single-center sample limit the causal inference and generalizability of the results, and future multi-center, longitudinal studies with larger sample sizes and diverse cancer types are needed to verify the findings.

## Figures and Tables

**Table 1:** Participant characteristics (N = 200).

Category	N/M	Percentage/SD
	N	N%
**Gender**		
Male	145	72.5%
Female	55	27.5%
**Marital status**		
Unmarried	10	5.0%
Married	188	94.0%
Divorced	2	1.0%
**Education level**		
Primary school and below	41	20.5%
Junior high school	86	43.0%
High School/Secondary vocational school	37	18.5%
Junior college	32	16.0%
Bachelor’s degree or higher	4	2.0%
**Family history**		
Yes	77	38.5%
No	123	61.5%
**Tumor stage**		
III	41	20.5%
IV	159	79.5%
**Types of cancer**		
Lung cancer	73	36.5%
Esophagus cancer	64	32.0%
Others	62	31.0%
**Religion**		
Yes	2	1.0%
No	198	99.0%
**Treatment methods**		
Chemotherapy	128	64.0%
Radiotherapy	89	44.5%
Targeted therapy	53	26.5%
Supportive care only	32	16.0%
**Scale Scores**	**M**	**SD**
**SRFS**		
SF1	3.16	1.14
SF2	3.00	0.90
SF3	2.81	0.72
**DCS**		
D1	1.99	1.22
D2	2.01	1.18
D3	1.24	0.63
**FACT-G**		
F1	2.10	1.11
F2	2.60	1.06
F3	2.55	0.92
F4	2.09	1.30

**Table 2:** Summary of core network indicators for each dimension.

Variable	Dimension Meaning	Strength	Closeness	Betweenness	Expected Influence
SF1	Cognitive Fatigue	0.779	0.014	12	0.326
SF2	Emotional Fatigue	1.444	0.015	30	−0.291
SF3	Behavioral Fatigue	0.670	0.011	0	0.353
D1	Uncertainty	1.202	0.013	4	0.274
D2	Decision-Influencing Factors	0.469	0.010	0	−0.223
D3	Effective Decision-Making	0.720	0.010	0	−0.469
F1	Physical Function	0.689	0.011	0	−0.004
F2	Social/Family Function	1.322	0.014	18	0.041
F3	Emotional Function	1.325	0.015	14	−0.302
F4	Functional Status	0.763	0.012	0	0.523

**Table 3:** Different subgroup network node core indicator differences.

Grouping Variable	Group Category	Key Node	Core Index	Index Value
Gender	Female	SF2 (Emotional Fatigue)	Bridge Strength	1.023
Gender	Male	F4 (Functional Status)	Expected Influence (EI)	0.541
Age Group	≤60 years old	SF1 (Cognitive Fatigue)	Expected Influence (EI)	0.352
Age Group	>60 years old	F2 (Social/Family Function)	Bridge Strength	1.156
Cancer Type	Lung Cancer	F4 (Functional Status)	Expected Influence (EI)	0.538
Cancer Type	Lung Cancer	D1 (Uncertainty)	Bridge Strength	0.889
Cancer Type	Esophageal Cancer	F2 (Social/Family Function)	Bridge Strength	1.132
Cancer Type	Esophageal Cancer	F4 (Functional Status)	Expected Influence (EI)	0.502
Cancer Type	Other Cancers	F4 (Functional Status)	Expected Influence (EI)	0.486
Treatment Method	Chemotherapy	SF3 (Behavioral Fatigue)	Expected Influence (EI)	0.367
Treatment Method	Supportive Care	SF2 (Emotional Fatigue)	Bridge Strength	1.012

## Data Availability

The data presented in this study are available on request from the corresponding author. The data are not publicly available due to privacy and ethical restrictions. Clinical resource: International Agency for Research on Cancer, https://gco.iarc.fr/.
